# Challenges and advances in the heterologous expression of cellulolytic enzymes: a review

**DOI:** 10.1186/s13068-014-0135-5

**Published:** 2014-10-18

**Authors:** Camilla Lambertz, Megan Garvey, Johannes Klinger, Dirk Heesel, Holger Klose, Rainer Fischer, Ulrich Commandeur

**Affiliations:** Institute for Molecular Biotechnology, RWTH Aachen University, Worringerweg 1, 52074 Aachen, Germany; Present address: School of Medicine, Deakin University, CSIRO Australian Animal Health Laboratory, 5 Portarlington Rd, Newcomb, VIC 3219 Australia; Present address: Institute for Botany and Molecular Genetics, RWTH Aachen University, Worringerweg 3, 52074 Aachen, Germany; Fraunhofer Institute for Molecular Biology and Applied Ecology, Forckenbeckstrasse 6, 52074 Aachen, Germany

**Keywords:** Cellulase, Heterologous expression, Cellulosome, Consolidated bioprocessing, Industrial processing

## Abstract

Second generation biofuel development is increasingly reliant on the recombinant expression of cellulases. Designing or identifying successful expression systems is thus of preeminent importance to industrial progress in the field. Recombinant production of cellulases has been performed using a wide range of expression systems in bacteria, yeasts and plants. In a number of these systems, particularly when using bacteria and plants, significant challenges have been experienced in expressing full-length proteins or proteins at high yield. Further difficulties have been encountered in designing recombinant systems for surface-display of cellulases and for use in consolidated bioprocessing in bacteria and yeast. For establishing cellulase expression in plants, various strategies are utilized to overcome problems, such as the auto-hydrolysis of developing plant cell walls. In this review, we investigate the major challenges, as well as the major advances made to date in the recombinant expression of cellulases across the commonly used bacterial, plant and yeast systems. We review some of the critical aspects to be considered for industrial-scale cellulase production.

## Reasons for interest in recombinant cellulases

Cellulases are a crucial component of various industrial processes, such as in cotton and paper manufacturing, as detergent enzymes, in juice extraction and as animal feed additives [1]. Moreover, cellulases are gaining more and more interest for agriculture, biotechnology and bioenergy uses [2], especially in the utilization of cellulosic biomass for the production of renewable liquid biofuels like ethanol, butanol or other fermentative products of sugar. With such uses, cellulases have the potential to become the largest group of industrially-used enzymes worldwide [3].

Cellulases hydrolyze cellulose, one of the three main components of lignocellulose (which also contains lignin and hemicellulose), into sugar molecules. Lignocellulose forms the cell wall and structural tissue of almost all plant systems. It is the most abundantly available regenerative raw material worldwide and, thus, plays an important role as a substrate in the conversion of biomass to biofuels. For the efficient hydrolysis of cellulose, basically three types of synergistically acting enzymes are necessary [4]. Cellobiohydrolases (CBHs), also named exoglucanases, attack the crystalline ends of cellulose producing cellobiose [5]. Endoglucanases (EGs) split glycosidic bonds within the amorphous part of the substrate [6]. Finally, the released cellobiose is cleaved by β-glycosidases (BGls) into glucose monomers [7,8].

Cellulolytic enzymes are widespread in nature and are found in plants, insects, bacteria and fungi [9-11]. Aerobic and anaerobic bacteria are known to produce cellulolytic enzymes as single enzymes or in the form of cellulosomes, multi-enzyme complexes comprising several cellulolytic enzymes bound to a scaffold protein [12]. However, the most common commercially available cellulases so far are non-complexed native enzyme mixtures derived from fungi, especially *Trichoderma* or *Aspergillus* species. For use as enzyme producers, cellulolytic fungi have the great advantage of both utilizing secretory pathways and the production of high protein yields [2]. Upwards of 20 g, and reportedly up to 100 g, of crude cellulases per liter are reachable with engineered *Trichoderma reesei* strains [3,13]. Additionally, other fungi, such as the genera *Penicillium*, *Acremonium* and *Chrysosporium* are viewed as potential and promising alternatives to *Trichoderma* [14].

For the conversion of biomass to biofuels on an industrial scale, several obstacles need to be overcome. For example, continued high production costs of cellulases, which comprise up to 20% of the total ethanol production costs as evaluated by the United States National Renewable Energy Laboratory (NREL) [2], minimize production efficiency on a commercial level. Further, to achieve efficient biomass conversion, a set of different enzymes have to act in concert, with the most effective enzyme composition being dependent on the respective biomass substrate [15,16]. The use of traditional fungal host organisms for cellulase degradation is restricted by the need for special culturing and induction conditions. To overcome these limitations, researchers are not only working on increasing the expression level of cellulolytic fungal cellulases to lower the production costs, but also on the optimization of recombinant expression systems in plants or microorganisms. The latter will allow for creation of microbial strains which express adapted, synergistically active sets of enzymes, either within a single cell [17] or by combining different strains [18], leading to enzyme yields on an economically feasible level.

This review will give an overview of the major problems and challenges of efficient recombinant expression of cellulolytic enzymes. The main focus will be on the differences between the most commonly used host organisms, whether in bacteria, yeast or plants. In particular, focus will be given to the less published challenges encountered, such as cases of enzyme truncation and expression failure of cellulase enzymes, thought to be due to problems occurring during the transport of proteins across the cell wall or intrinsic host-specific mechanisms.

## Cell wall features and protein transport of commonly used host organisms

**Figure 1:**
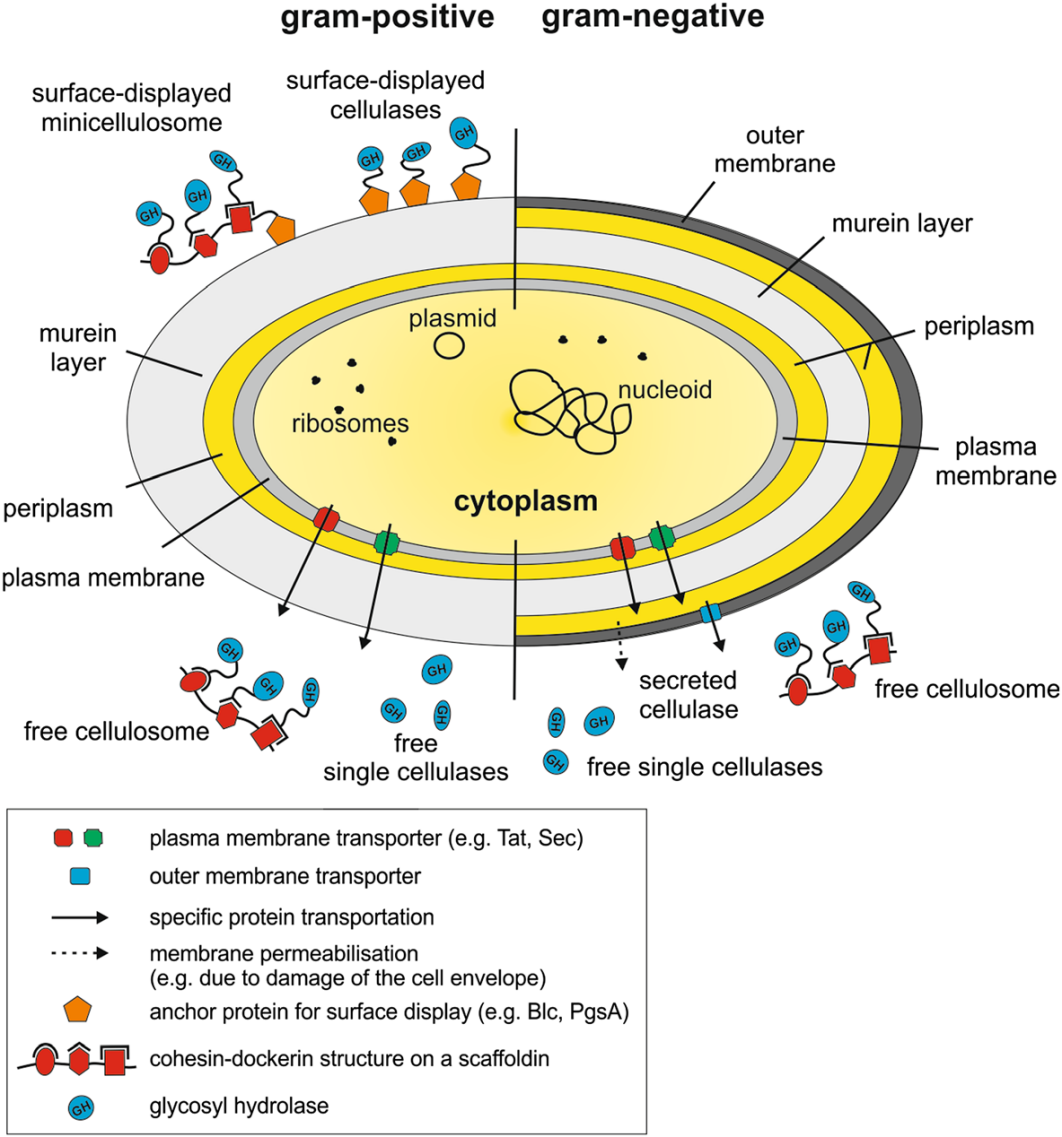
**Schematic drawing of a bacterial cell showing the localization of recombinantly expressed cellulases and cellulosomes.** Recombinant cellulases are expressed and stored in the cytoplasm or targeted to the periplasm through specific protein transporters (such as Tat- and Sec-pathways). Gram-negative bacteria (right) possess a thick outer membrane that restricts extracellular protein transport, thus protein secretion occurs via a specific protein transporter or membrane permeabilization (occurring, for example, due to damage of the cell envelope). Gram-positive bacteria (left) lack the outer membrane leading to a more efficient secretion. Secreted cellulases are either free in solution, either as single cellulases or as cellulosomes, or are displayed on the cell surface via an anchor protein such as Blc or PgsA as single cellulases or as mini-cellulosomes. GH: glycosyl hydrolase; Sec: secretion protein; Tat: twin-arginine-transporter.

**Figure 2:**
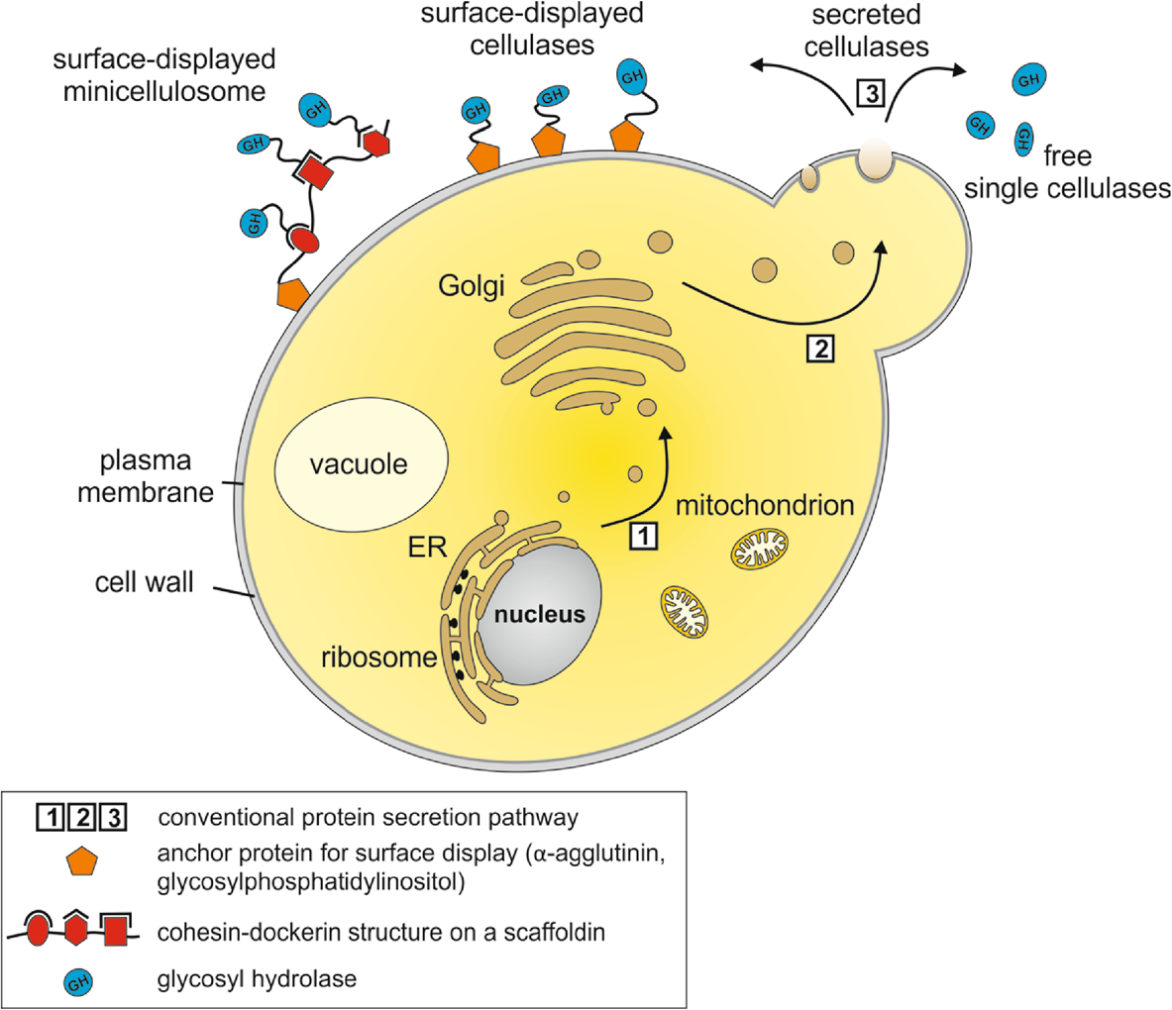
**Illustration of a single yeast cell showing the main cell compartments involved in recombinant protein expression.** Arrows indicate the secretory pathway, whereby cellulases are expressed on the ER (1) and transferred via the Golgi apparatus (2) to the medium (3) in secretory vesicles. Cellulases are either free in solution or surface-displayed via an anchor protein (such as α-agglutinin) as single cellulases or as mini-cellulosomes. ER: endoplasmic reticulum; GH: glycosyl hydrolase.

**Figure 3:**
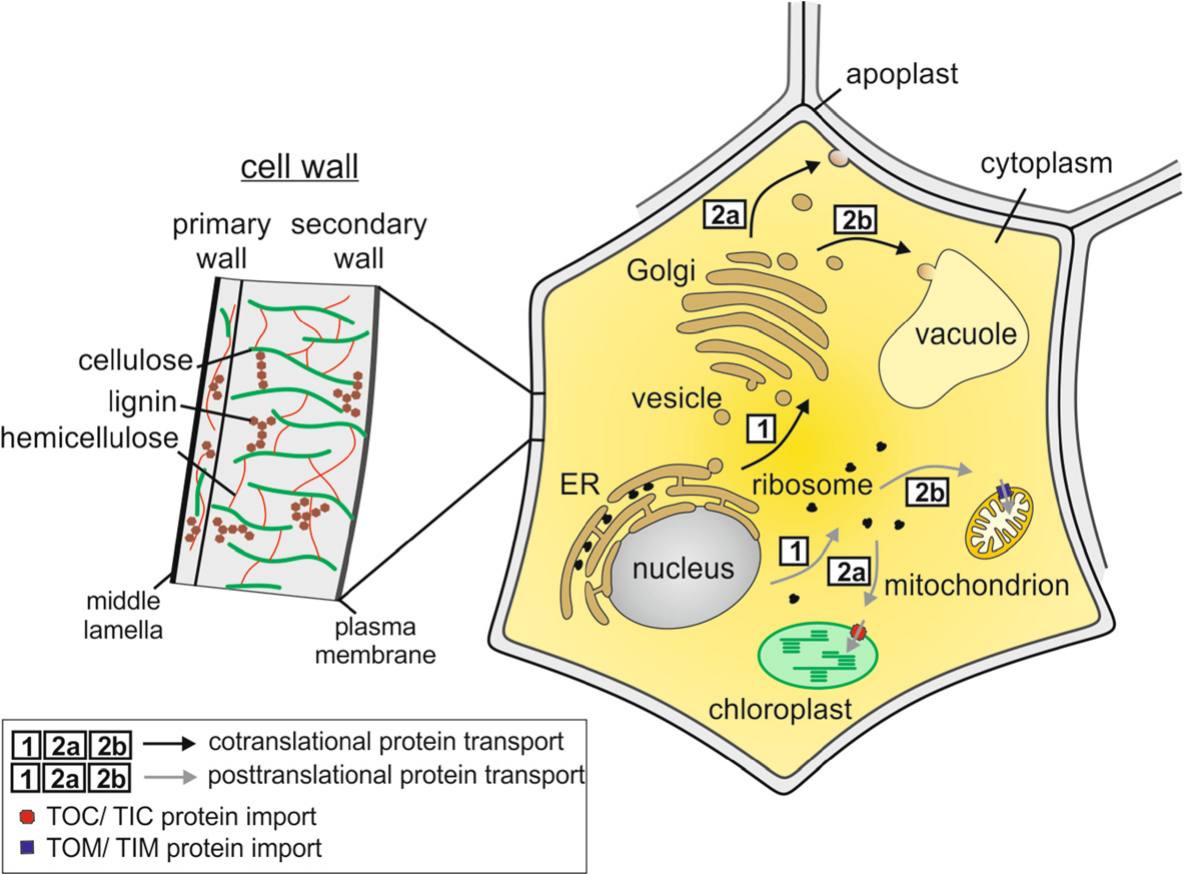
**Plant cell drawing with the main cell compartments for recombinant protein expression and targeting.** Right: Nuclear encoded genes are expressed and targeted to different subcellular compartments using one of two different pathways, indicated by black or grey arrows. On one hand, translation occurs directly into the ER lumen by ribosomes associated with the ER. From here, proteins are transferred into vesicles via the Golgi apparatus (black arrow (1)) to the apoplast (black arrow (2a)) or vacuole (black arrow (2b)) and secreted. Other gene transcripts are translated by free ribosomes (grey arrow (1)) and targeted to the chloroplast (grey arrow (2a)) or mitochondrion (grey arrow (2b)) via specific transit peptides and protein import mechanisms (grey arrows). Left: Enlarged section of the plant cell wall which is primarily composed of cellulose, lignin and hemicellulose. ER: endoplasmic reticulum; TOC/TIC: translocon at the outer envelope of chloroplasts/translocon at the inner envelope of chloroplasts; TOM/TIM: translocase of the outer mitochondrial membrane/translocase of the inner membrane.

To date, many different host organisms are used for cellulase expression, including bacteria (such as *Escherichia* and *Clostridium* species), yeast (such as *Saccharomyces* and *Kluyveromyces* species) and plants (such as maize and tobacco). These organisms differ significantly in their cell wall structure, the subcellular compartments for protein targeting and their protein secretion possibilities (Figures 1, 2 and 3).

Bacteria are divided into gram-positive and gram-negative bacteria, depending on their cell wall features. Gram-positive bacteria (like *Clostridium thermocellum*) use protein-secretion mechanisms, including the twin-arginine transporter (Tat) and secretion (Sec) pathways (Figure 1), which export proteins across the cytoplasmic membrane [19]. However, gram-negative bacteria (like *Escherichia coli* and *Zymomonas mobilis*) possess an additional outer membrane which restricts the extracellular transport of proteins. Therefore, gram-negative bacteria utilize a variety of secretory pathways for cellulase secretion [20], contributing to the challenges in engineered cellulase production [21].

In contrast to prokaryotic cells, eukaryotic cells are characterized by a nucleus and a variety of organelles. The cells are surrounded by a double lipid layer with a varied composition depending on the organism and its individual function. Yeast cell walls feature a three-layered organization. The inner plasma membrane is covered by an electron transparent layer of glucans, which is complexed with a minor component of chitin, and an outer layer consisting mainly of mannoproteins, which are covalently linked to the inner glucan layer [22]. Similarly to higher eukaryotic cells, protein secretion is directed by an amino-terminal signal peptide sequence which mediates cotranslational translocation into the endoplasmic reticulum (ER), where the signal peptide is removed. N-linked oligosaccharides may be added when the protein is transported via vesicles from the ER to the Golgi, where further modification of these glycosyl structures occurs. From here, the proteins are transferred to the cell surface inside secretory vesicles for secretion (Figure 2).

The plant cell wall differs in composition and thickness according to plant age, species and tissue [15,23]. A schematic drawing showing the different layers of a plant cell is given in Figure 3. Proteins can accumulate either within the cytoplasm, by targeting via specific signal and transit peptides to different subcellular compartments (the chloroplast, the mitochondria and the ER) or being transported within secretory vesicles from ER through the Golgi network to the vacuole or apoplast [24]. This protein secretion pathway is similar to the above-mentioned yeast secretion pathway, although in plants no hyperglycosylation of proteins occurs [24].

## Bacterial expression systems

A wide range of actinomycetes (such as *Thermobifida fusca*, *Thermomonospora curvata* and *Cellulomonas fimi*) [25-27], *Bacillus* species [28] and *Clostridia* species [29] are cellulase-secreting organisms [20]. Expression of cellulases relies on induction of the relevant genes, therefore these proteins are best expressed when these microbes are grown on cellulosic substrates with no easily metabolizable carbon sources [25-29]. The optimal cellulosic inducer varies from organism to organism, and even the most successful induction-associated cellulosic substrates, such as cellobiose and carboxymethyl cellulose (CMC), feature organisms which fail to respond with cellulase production [25]. Not just the carbon source but also the nitrogen and phosphorous sources have been reported to have a significant impact on homologous cellulase expression [27].

Heterologous expression of cellulases can also be triggered in rich growth media by the use of inducible or auto-inducible promoters. For example, the heterologous expression of an alkaline cellulase in a cellulolytic *Bacillus* strain was achieved under control of a sucrose inducible sacB promoter via the use of a rich growth medium containing yeast extract, tryptone and glucose. Using this system, the expression level of the heterologous cellulase was raised 20-fold, as compared with expression under its own promoter [30]. In another study, a β-galactosidase was expressed in a *Bacillus* strain under control of an artificial stationary phase specific, auto-inducible promoter, based on an engineered cry3Aa promoter sequence [31], which may also be applicable for cellulase expression. A two-fold increased CelA2 protein production in *E. coli* was achieved using epMEGAWHOP, a method by which the vector backbone is randomly mutated [32].

The most frequently used bacterial expression host in laboratorial studies is *E. coli* [33], due to its well-characterized and easy-to-manipulate genetics, the abundance of commercially-available strains and vectors and its ability to express recombinant genes in high yields of up to 50% of the total protein [34]. However, attempts at the expression of cellulases in *E. coli* have encountered numerous problems such as degradation of linker sequences in multi-domain cellulases [35-37], formation of inclusion bodies [38,39], incorrect transportation across the outer membrane [40] and decreased specific activity of the cellulases [41] (Table 1).

**Table 1:** **Advantages and disadvantages of homologous and heterologous protein expression in different host organisms in regard to technical, ethical and economic aspects**

**Organism**	**Example**	**Protein expression**	**Advantages**	**Disadvantages**	**Expression challenges**
Homologous cellulase production systems					
Fungi	*Trichoderma reesei*	14 000 to 19 000 mg/l crude enzyme solution [79]	Native system	Enzyme mix cannot be tailored for different biomass substrates	Special culturing conditions required
			Protein secretor	Comparably high production costs	
			High protein yield		
Bacteria	*Bacillus subtilis* (gram positive)		Inducible and auto-inducible expression possible	Rich growth medium required as a carbon source, leading to increased costs	Inducible systems more efficient but significantly more expensive
			Easy to modify genetically		
			Protein secretor		
	*Clostridium thermocellum* (gram positive)		Native system	Low protein yield	Special culturing conditions required
			Cellulosome producing	High production costs	
			Transient and stable transformation	Unwanted byproducts	
Heterologous cellulase production systems					
Bacteria	*Escherichia coli* (gram negative)	11.2 to 90 mg/l purified enzyme solution [79]	Industrially used, common system	Thick outer membrane restricts protein secretion (poor secretion)	Degradation of linker sequences in multi-domain cellulases
			Well-characterized genetics		Formation of inclusion bodies
			Many commercially available strains and vectors		Frequently incorrect transportation across the outer membrane
			Easily to modify for example for protein engineering		Decreased specific activity of the cellulase can occur
Yeast	*Saccharomyces cerevisiae*	Approximately 1 000 mg/l crude enzyme solution [79]	Protein secretor	Hyperglycosylation	Inducible systems are highly efficient but can be expensive
			Surface display possible	Expression rates lower than native systems	Increased episomal gene copy numbers leads to higher protein yields but a constant selection is necessary
			Industrially used, common system		
Plants	*Nicotiana tabacum*	Up to 40% of total soluble protein, depending on the subcellular targeting inside the plant cell [79]	Cheap protein production	Transport of genetic information via pollen (if not transplastomic)	Possible glycosylation effects
			Easy transformation	Long transformation procedure	Subcellular targeting inside the plant cell very important for expression efficiency
			Well-characterized genetics		Possible effects on plant growth behaviour
			Protein and biomass		
			Production in one system		
			Non-food		
	*Zea mays*	Approximately 0.45% of dry weight [159]	Cheap protein production	Transport of genetic information via pollen (if not transplastomic)	Possible glycosylation effects
			Simultaneous biomass and enzyme production	Long transformation procedure	Subcellular targeting inside the plant cell very important for expression efficiency
			System already used for biofuel production	Food	

Further, more general limitations of *E. coli* as an organism for heterologous cellulase expression are low production levels of soluble proteins and poor secretion ability. Different approaches have been employed to overcome the insolubility of heterologously expressed proteins in *E. coli*. The soluble expression of previously insoluble cellulases has been achieved in *E. coli* by strategies such as the fusion of a selected catalytic domain to the carbohydrate-binding module (CBM) of another soluble cellulase [39]. Other strategies are based on the purification of cellulase-containing inclusion bodies using agents such as urea and β-mercaptoethanol with subsequent refolding [42,43], or on the reduction of the expression temperature to ensure improved protein folding.

One example of temperature-related expression variability in *E. coli* is the cellulase from the springtail of *Cryptopygus antarcticus*, which is best expressed as a soluble protein at 10°C [44]. Another potential problem is the low secretion of recombinant cellulases in *E. coli*, which can be improved by ethylenediaminetetraacetic acid (EDTA) treatment [45] or by the expression of cellulases as OsmY fusion proteins [46]. OsmY is induced by hyperosmotic stress [47] and supports the transport of the fusion proteins over the outer membrane [46], where the fused protein gets cleaved off. Co-expression of the cellulase CelZ and proteins, belonging to the SEC pathways of *Erwinia chrysanthemi*, facilitate the secretion of CelZ in *E. coli* [48] and the secretion of *E. chrysanthemi* EG CleY and CelZ in *Klebsiella oxytoca* [49], respectively. Further, it was found that the deletion of Braun’s lipoprotein makes the outer membrane of *E. coli* more permeable, which resulted in enhanced secretion of periplasmic proteins and was shown to elevate the overall expression rate of an EG [50].

Another important consideration when expressing cellulases in *E. coli* is the strain selected for expression. Factors such as whether a strain enables disulfide bridge formation in the cytoplasm (like *E. coli* Rosetta-gami™) appear to have a significant impact on the expression of certain cellulases [44]. Cellulolytic expression can also be affected by folding factors, with incorrect folding being linked to significant reduction of activity in recombinant proteins compared to their correctly folded counterparts [51]. Lastly, the ongoing optimization of *E. coli* for recombinant protein expression, for example increasing the ability of secretion via different approaches [34] or the functional transfer of N-linked glycosylation into *E. coli* [52] is very promising for enhanced cellulase production in this organism since it is assumed that the glycosylation pattern of the linker region of the cellulase is responsible for the flexibility [53] and protease resistance [54] of the enzyme.

### Surface-displayed cellulases, chimeric cellulases and cellulosomes

Bacterial expression systems also show great promise in the creation of chimeric cellulases and cellulosomal systems. Chimeric cellulases can be designed to heighten the activities of cellulases by tethering two different activities together [55,56], however they are more commonly created as components for cellulosomes or mini-cellulosomes [57-61]. Bacterial strains used for the expression of these components have included *E. coli* [62-64], but gram-positive *Bacillus subtilis* or *Clostridium* species are preferentially used due to their stronger secretion abilities, surface display properties and already being cellulosome-capable [62,65].

In *B. subtilis* a number of mini-cellulosomes, or the components to create mini-cellulosomes, have been successfully expressed, containing a wide variety of enzymes, enzyme-binding domains and enzyme-catalytic domains, derived from bacteria such as *T. fusca* [66,67] or *Clostridium* species [62,68-70]. To cite only a few further examples, *Clostridium cellulovorans* proteins have also been expressed in butanol, acetone and ethanol producing *Clostridium acetobutylicum*, however with low yields [71]. Mini-cellulosomes with *C. thermocellum* components have been produced in the industrially-used *Lactococcus lactis* [72] and in *Corynebacterium glutamicum*, which can also be used for the production of amino acid products [73]. Cellulosomal components primarily created from cellulase and hemicellulase genes originally found in *C. cellulovorans* and *Clostridium cellulolyticum*, but also from such varied sources as *Aspergillus niger* and *Neocallimastix patriciarum*, have been successfully expressed in *E. coli* for *in vitro* assembly and used either as a complete system [42,43,74], in combination with *B. subtilis* produced components [62] or in systems using nanoparticles as the cellulosome scaffold [63,64].

The (mini-)cellulosomes described above can be separated into four distinct categories: (1) free in solution, (2) complexed in solution, (3) surface-displayed and (4) mixtures thereof (Figure 1). The critical prerequisite for soluble cellulosomes is that the proteins are properly expressed and secreted. This is not always a simple requirement, as described above. Those enzymes to be complexed in solution require expression of the enzymes of interest, which need to contain a dockerin or other binding module, as well as the expression or presence of a chosen scaffold, such as a protein cohesin or other binding modules [64,75,76]. For the production of these heterologous proteins, native secretion mechanisms are most frequently used [73]. The third category, the creation of surface-displayed cellulosomes, requires native secretion or fusion mechanisms as well, however heterologous systems, such as *Ruminococcus flavefaciens* [69] and *Streptococcus pyogenes* [72], have also been utilized. Preliminary investigations have been performed examining the surface display of individual cellulase proteins in *E. coli*, both via lipoproteins, as a proof of principle [77] and via ice nucleation proteins from *Pseudomonas syringae* for the creation of a screening library to identify improved cellulase variants [78].

### Application-related progress using bacteria

Recombinant expression of cellulase enzymes in bacterial systems has proven to be difficult and a number of key challenges remain to be overcome (reviewed in Garvey *et al*. [79]). However, over the past few years, increasing understanding of the problems faced has led to significant progress in the expression of both single cellulase enzymes and in the creation of cellulase mixtures and cellulosomes in bacteria. There are several reports where a homologous set of cellulases, such as those in *B. subtilis*, have been supplemented by the introduction of heterologous cellulase-coding sequences [30,80,81]. Additionally, non-natively cellulolytic bacteria, like *E. coli* and *Z. mobilis* have been transformed into cellulolytic bacteria by genetic manipulation, in order to achieve production of special metabolic products using cellulosic substrates as their carbon source [82,83] or to enhance the properties of probiotic microorganisms [84]. For example, a carboxymethyl cellulase from the marine *B. subtilis* was heterologously expressed in *E. coli* in a 7-liter bioreactor showing a 5.9-fold higher activity rate than in its native organism [85]. Although no clear forerunner for a bacterial consolidated bioprocessing (CBP) organism has yet been established, the tractability of bacteria for protein engineering and transformation into cellulolytic organisms makes this a highly likely prospect for future success [86].

## Yeast expression systems

The majority of cellulases are expressed via secretory pathways in their native organisms. When expressed heterologously in *E. coli* they are, as discussed above, frequently insoluble due to the intrinsic nature and intracellularly-focused expression systems of this bacterium. Yeast, as a host organism, is known for high expression yields in the range of grams per liter [87,88] and the above-mentioned enzymes are likely to be expressed in soluble and active forms. The secretory pathway of yeasts enables the formation of favored disulfide bonds as well as glycosylation, enhancing the stability and potentially the functionality of the enzymes [89]. Glycosylation of the enzyme linker regions, which typically connect the catalytic domain and the CBM of cellulases, provides protease protection [90] and may also play an important role in assisting the binding of the cellulase to the cellulose surface via the CBM [91]. Further, tractability and easy cultivation conditions are important aspects. This opens the possibility of protein engineering, supported by computational studies (a successful method for cellulase optimization [92]), such as the creation of more active and thermostable enzymes [93].

High throughput approaches, such as flow cytometry and microfluidics-based methods, are also being utilized to identify highly active cellulases, with the potential for use in selecting enzymes from existing or new variant libraries [94]. Another reason for the increasing interest in yeast systems for heterologous cellulase production is their potential for displaying enzymes and enzyme complexes on the surface. Yeast hosts have been demonstrated to be capable of expressing multiple free cellulases within single-cell yeast strains, such as *Saccharomyces cerevisiae* [95], *Saccharomyces pastorianus* [96], *Pichia pastoris* [97,98] and more recently the *Kluyveromyces marxianus* [99]. Surface display of cellulase proteins has also been shown in yeast, both for single enzymes [100], multiple enzymes [101] and for (mini-)cellulosome production (see below) (Figure 2). It is notable that, unlike in many bacterial systems, the inherent protein secretion and display mechanisms of yeasts work successfully with cellulase proteins; examples include the use of *S. cerevisiae* α-mating factor as a secretion signal and α-agglutinin as an anchor protein [82]. Lastly, established tools for genome manipulation and easy cultivation conditions are important aspects for choosing yeasts as an expression host organism.

The expression and secretion of many cellulase-encoding genes in yeast strains have been reported so far and tested on a diversity of substrates, including synthetic compounds as well as amorphous and crystalline forms of cellulose (reviewed in den Haan *et al*. [102]). Highly efficient secretion was observed by using the yeast α-mating factor for different strains instead of the formerly used yeast signal sequences PHO5 [103] and SUC2 [104]. Overexpression of multiple native SNAREs (soluble NSF (N-ethylmaleimide-sensitive factor) attachment receptor proteins), which play an important role during intracellular membrane transport, can increase secretion of heterologously expressed CBH and BGl in *S. cerevisiae* by up to 52% [105]. Further optimization by random mutagenesis of an EG gene expressed in *S. cerevisiae* resulted in increased thermostability and extracellular activity [106]. A higher expression yield of CBH was achieved by structure-guided SCHEMA recombination [107] and a 4.5-fold increased *Saccharomycopsis fibuligera* BGl1 production was realized by the simultaneous overexpression of native *S. cerevisiae* PSE1 (encoding for a karyopherin) and SOD1 (encoding for a superoxide dismutase). Both proteins were identified through a polygenic analysis for the identification of secretion-enhancing genes [108].

Using δ-integration, a method by which the delta sequence of yeast retrotransposons Ty is used for a multi-copy integration [109], in combination with different auxotrophic markers influences the expression of cellulases significantly, which might be helpful to control the expression of exogenous genes, especially the co-expression of multiple genes [110]. Beside the knowledge of the positive impact of promoter design [111] and (synthetic) terminator regions for mRNA stability and activity [112], an artificial transcriptional activator (TA) was expressed together with an expression cassette containing multiple TA-binding sites and one of three secretory cellulases. An optimal combination of the number of TA-binding sites, the type of core promoter and the terminator region leads to 1.5 to 4-fold increased secretion and 1.5 to 2.5-fold increased activity against phosphoric acid swollen cellulose (PASC) [113]. Looking at other yeast systems, the expression of high yields of foreign cellulases with comparable activity and stability to the native forms were reported in *P. pastoris* [114-116], where a positive effect by codon optimization could be shown [114]. Recently, a *P. pastoris* strain, based on the commercially available PichiaPink™ expression system, was optimized for the constitutive EG production from *Aspergillus* at bioreactor scale, which yielded up to 5 g/l total secreted protein [88]. Great progress has also been made for organisms from the species *Kluyveromyces* [34,99,117]. Chang *et al*. transformed *K. marxianus* simultaneously with a five-gene cassette, including a CBH, a EG and a BGl, leading to a β-glucan-into-ethanol converting strain [34]. This strain has since been improved by the addition of further cellulases and a cellodextrin transporter gene into the genome, leading to direct conversion of cellulose to ethanol by the recombinant strain [99].

### Cell surface display and cellulosomes

The production, secretion and display of cellulolytic proteins as (mini-)cellulosomes in yeast hosts are a significant aim of current research [61]. The generation of such large, complexed cellulase mixtures would help to offset the significantly lower protein expression rates in yeast systems as compared to fungal systems. An economically feasible proposition for future fuel generation is the use of large cellulosomes with significant saccharification capacity to be displayed on a CBP-capable organism [59], with a number of yeasts being viable options. Towards this goal, (mini-)cellulosomes have been developed within *S. cerevisiae* both as consortia, where enzymes and scaffold proteins are grown in individual strains [118], or as multiple proteins expressed within a single strain [119]. Variations on the use of the α-mating factor secretion pathway were again used to enable successful protein secretion. Attachment mechanisms included α-agglutinin and glycosylphosphatidylinositol anchors [18,118] as well as systems inherent to the species [119]. A cellulosome consisting of a clostridial scaffoldin, carrying an EG from *Nasutitermes takasagoensis* was expressed in *P. pastoris* and linked to the cell surface via a lectin-like anchor flocculation protein from *S. cerevisiae* [92]. Non-surface associated mini-cellulosomes have also been successfully created via the use of yeast secretion mechanisms, which would provide many benefits of cellulosome activity without the difficulties involved in scaffold attachment (Figure 2) [120].

### Challenges and drawbacks in cellulase expression and secretion in yeast

Despite the above-mentioned progress, many challenges still lie ahead for economically viable expression of individual cellulases in yeast systems (Table 1). Factors yet to be determined include the reason why CBHs with high homology are secreted at significantly different levels, and why the co-expression of multiple enzymes in one cell leads to decreasing levels compared to their expression in separate single-cell systems [102]. Different molecular biological approaches to overcome this issue have been inconclusively discussed in the literature, such as promoter design or multi-copy integration of the gene sequence, whether integration is episomal or genomic [96]. Using *S. cerevisiae*, Ilmen *et al*. suggested a ’compatibility factor’, characterized by different levels of plasmid, mRNA and secreted proteins, to explain how some CBHs are more compatible with high level expression and production than others [121]. Variations in the unfolded protein response induction indicate different levels of cellular stress by the expression of different cellulases. This response is suggested to be a novel type of feedback mechanism called repression under secretion stress (RESS) that is activated in response to an impairment of protein folding and secretion. To avoid RESS, the authors recommend using constitutive glycolytic yeast promoters during heterologous expression [102].

During the multi-step cellulase secretory process a number of problems may occur. Yeast proteins which assist in folding and disulfide bond formation differ between organisms, affecting the folding of foreign proteins. Misfolding can result in degradation or retention in the ER and Golgi, respectively [122], and the formation of improper disulfide bonds are one possible reason for the small percentage of active enzyme produced, for example in relation to the secreted fraction in the case of *T. reesei* CBH1 expressed in *P. pastoris* [123]. Alternatively, expression of *Chaetomium thermophilum* CBH3 was produced actively in *P. pastoris*, suggesting that better expression rates may be achievable when using proteins which consist only of single catalytic domains, rather than the more frequent three-domain proteins (containing a catalytic domain, linker and carbohydrate-binding domain) [124]. Another point which has to be taken into consideration is the specific glycosylation provided by each organism. Expression of a protein in a heterologous system will most likely result in a product with different posttranslational modifications compared with the native enzyme. *S. cerevisiae*, for instance, lacks the Golgi mannosidase which is present in higher eukaryotes, leading to an elongation of the oligosaccharide chains of the recombinant enzymes by adding further mannose residues [122]. Thus, extensive and branched chains are built up which may add benefits such as protease resistance and thermostability or, alternately, may impair correct protein folding, reactivity and proper secretion.

### Application-related progress

One challenge is the creation of yeast organisms combining cellulolytic and fermentative capacities. Previously, strains have been created which could convert cellobiose [125], amorphous cellulose [126] and even crystalline cellulose [121,127], at least partially, to ethanol. Traditionally, *S. cerevisiae* was chosen for such approaches, including transformation by cellulase genes and improvement of protein secretion. However, no economically feasible CBP organism has so far been created, despite over a decade of work towards this goal [128,129]. Direct ethanol fermentation was shown for *S. cerevisiae*, which co-expresses several surface-displayed cellulases and a cellodextrin transporter, but the ethanol production rates were far behind industrial production levels [130]. More recent approaches utilize alternative yeast organisms with the hope of greater success at establishing an economically viable CBP organism. For example, *Kluyveromyces* sp. secretes proteins more efficiently and with a less extended glycosylation pattern than *S. cerevisiae* [131,132]. This, together with its broad growth temperature and pH ranges, makes it a promising candidate for CBP [99]. As mentioned above, direct conversion of cellulose into ethanol has been shown for a recombinant *K. marxianus* strain, which expressed and secreted five cellulases and one cellodextrin transporter simultaneously [99]. However, these results affirm the potential of *K. marxianus* and *S. cerevisiae* as future CBP strains. Separate hydrolysis and fermentation *in situ*, another possible approach, is focused on the idea of a two-step approach, a pre-hydrolysis of the cellulose substrate followed by fermentation, which enables optimal conditions for each reaction step [96].

## Plant expression systems

The most obvious benefits of cellulase production in plants are low production costs and the ease of achievable scale-up. Both are crucial factors for the economically feasible production of lignocellulosic biofuels due to the high amounts of enzymes needed during the process [133] (Table 1).

### Cellulase expression in different plant cell compartments

To achieve efficient cellulase expression in plants, differential targeting of the enzymes is an important strategy towards obtaining high yields and activity. Depending on the (sub)cellular compartment targeted, significant effects on enzyme stability and expression level have been observed [134]. Additionally, the ability of different compartments to allow posttranslational modification of cellulases is an important consideration, as this may impact on the enzymatic activity [135], dependent upon the source of the cellulase expressed (fungal or bacterial). One example of the effects caused by differential targeting is seen with the EG E1 from *Acidothermus cellulolyticus*, which showed low expression levels in the cytosol of several plant species. Higher yields of this enzyme, up to 16% of total soluble protein (TSP), were achieved when targeted to different subcellular compartments like apoplast, vacuole, ER or mitochondria [136-138]. Similar results have been shown for bacterial cellulase Cel5A from *Thermotoga maritima*. Whereas cytosolic protein expression in tobacco leaves failed, the enzyme accumulates up to 5% of TSP when targeted to the chloroplast, vacuole or apoplast [139]. Even higher amounts, up to 9.3% TSP, could be achieved by the expressing of BglB cellulase from *T. maritima* in the chloroplast of tobacco using a modified alfalfa *RbcsK-1A* promoter and a transit peptide [140]. This clearly indicates that the choice of the subcellular compartment plays an important role in heterologous cellulases expression in plant systems.

A second important factor in the expression of cellulase enzymes through plant systems is in the potential for truncation of the enzyme or enzymes. This is also somewhat dependent upon the cellulase localization inside the cell. Again, using the example of EG E1 from *A. cellulolyticus,* it has been observed that the full-length enzyme is mostly expressed in a truncated but active form, representing only the catalytic domain of the enzyme [134]. The expression of only the catalytic domain of E1 resulted in an increased protein yield, independent of the chosen cell compartments. Evidence even suggests that the truncated catalytic domain of E1 has equal or greater activity to the full-length enzyme [134], making this a beneficial alteration for this specific enzyme. However, for other cellulases this may not be the case, in particular it is known that CBHs need their CBMs in addition to the catalytic domain to achieve effective binding and processivity [141]. One alternative to dealing with the possible truncation of enzymes is to produce single domain enzymes, such as the endocellulase from the hyperthermophilic archeon *Sulfolobus solfataricus*, which has been successfully expressed in its active form in the ER of tobacco [142].

As an alternative to nuclear genome transformation, the chloroplast is another potential site for heterologous expression with reported yields up to 70% of TSP for a recombinant protein antibiotic [143]. Chloroplast transformation techniques have been utilized to increase the yield of *in planta* production. Thus, different bacterial cellulase enzymes, predominantly derived from *T. fusca*, were expressed in active form with yields from 2 to 40% of TSP, mostly without degradation effects [144-148]. Consequently, for heterologous expression of full-length bacterial cellulases, the prokaryotic environment of the chloroplast seems to be a very suitable site. Chloroplast transformation provides additional advantages like whole operon expression [149] of multiple genes and transgene containment via maternal inheritance [150,151].

The ER is another promising subcellular compartment for bacterial cellulases due to its chaperones, which lead to better protein folding and stability [152]. Eukaryotic cellulases expressed in plants are commonly derived from *T. reesei*, for which ER-targeted CBHs led to truncated but active enzymes, for example in maize [136]. The EGs from *A. cellulolyticus* and *S. solfataricus* (discussed above) are other examples for successful cellulase production in the plant ER.

There are indications that targeting to the apoplast may also provide good enzyme yields, but this is countered by possible detrimental effects on plant development, such as decreased growth and reduced cellulose content. To avoid these effects, inducible expression systems can be utilized such as the alcohol inducible promoter system described by Klose *et al*. [153]. However, recombinant cellulase expression can have far more complicated effects on the growth and development of the plants. Some studies even reported positive effects of heterologous cellulase expression on plant development, such as increased length and width of stems, as shown by Hartati *et al*. [154] who expressed a poplar cellulase in sengon wood to successfully improve the growth performance of this industrially-used species.

When expressing cellulases in plants, it is important to consider all factors concerning the expression levels and relative activity of the enzyme in relation to its localization within the plant. There is no current forerunner for the ‘best’ compartment for cellulase expression, with the variety of activities within the glycosyl hydrolase family and the differences in processing of enzymes from different sources making such general statements impossible. As a result, the heterologous expression of each enzyme will need to be examined independently for the effects on plant growth and development, as well as for enzyme yield and activity. Despite this, plant systems remain one of the most promising methods for cellulase production on a large and sustainable scale.

### Different plant sources for biomass utilization

Beside the research approaches in model systems like tobacco and *Arabidopsis thaliana*, expression of glycosyl hydrolases and especially cellulases has also been achieved in a variety of other plant species. Crops and woody plants are already implemented in certain industrial processes and are therefore promising candidates as production platforms for biomass-degrading enzymes like cellulases. The three most prominent world crops (maize, rice and wheat) have already been employed in transgenic glycosyl hydrolase production [155-157]. An example that is already commercially available is based on the utilization of starch, where a thermostable α-amylase is being expressed in transgenic maize. This maize, called Enogen™, is currently being utilized by Bonanza BioEnergy (Garden City, Kansas, United States) for the production of ethanol from starch [158].

For lignocellulosic fuel production, a transgenic crop producing cellulolytic enzymes to be utilized in an economical process has yet to be created. However, several studies have already described the heterologous expression of cellulolytic enzymes in maize with remarkable success in achieving high expression levels [136,155,159]. Amounts of 0.4 kg/ton maize stover are reported for *A. cellulolyticus* EG E1, and up to 1.8 kg/ton grain for *T. reesei* CBHI expressed in maize germplasm [138,159]. Apart from maize, cellulases have been successfully expressed in other crops, like rice [137,156] and sugar cane [160,161]. Studies on woody biomass expressing cellulolytic enzymes are rarer than those for crop plants. Nevertheless, successful expression of a recombinant EG has been produced in poplar plants [157,162]. In these studies, the impact of the recombinant EG on plant growth and development, especially the cell wall, was highlighted.

All approaches developed to date for producing fuel from lignocellulosic sources face a common challenge. The amount of enzyme produced remains significantly lower compared to the amount required for complete biomass degradation [163,164]. Hence, the possibility of modifying plant cell walls for improved biomass degradation is a promising addition to using plants purely for cellulase production. Here, the concept is that the *in planta* expression of enzymes leads to modification of the complex plant cell wall structure (Figure 3) and therefore a reduced recalcitrance of plant biomass during degradation for biofuel processing. An example for this strategy is the heterologous expression of glycosyl hydrolases, cellulases and xylanases, which have been shown to improve the hydrolysis rate of transgenic tobacco and maize [47,165,166], as demonstrated by Zhang *et al*., where transgenic maize expressing different biomass-degrading enzymes was utilized for hydrolysis with subsequent fermentation to ethanol, resulting in a 55% improved conversion of lignocellulose to ethanol compared to control plants [166]. For this reason, more recent studies often incorporate detailed information of cellulase effects on plant growth and development, such as the examination of recombinant EG activity on plant cell walls in the poplar studies mentioned above [157,162]. It must be noted, however, that for field application of these systems, the plant fitness and robustness will have to be considered. In particular, it would need to be determined if resistance to pathogens and herbivorous insects could be altered through such cell-wall modifications [167].

## Concluding remarks

The importance of identifying effective systems for recombinant cellulase expression will be one of the key factors in the success of second generation biofuel production. Enzyme production is a critical factor in the economic validation of these systems and a significant stumbling block in establishing systems at the current level of technology. The progress towards better cellulase expression systems, discussed in this review, reveals two major approaches to meeting the challenges within the field. Firstly, many researchers are using systems biology approaches to overcome the difficulties in the heterologous expression of these proteins, such as by the use of different promoters or sequence optimization to increase protein production, or through the use of bacterial or yeast species variants which provide different folding or posttranslational modification options, enabling higher yields of active protein. A second feasible approach to many of the challenges faced has been by moving from multi-domain cellulase expression to expression of single domain proteins (or those active when only containing a single domain), which removes the problem of low yields due to incorrect folding or processing.

While such strategies have progressed our understanding of recombinant cellulase production and have moved the field closer to a viable solution, large challenges remain before cellulase production yields and activities are high enough to be industrially relevant. Work towards meeting this challenge through enzyme surface display or cellulosome production remains far behind the necessary level for such systems to be implemented in the near future. Similarly, the attempts to establish CBP-capable systems continue to progress slowly, without even a clear lead organism in focus. Currently the most promising systems for cellulase production, as evidenced by commercial interest (other than modified homologous systems), are in plant systems for enzyme production. Here, differential targeting provides great promise for successful production of active cellulases, while the already established systems of large-scale plant farming allow for the simple implementation of new systems. However, the current political climate in many countries towards the use of genetically engineered plants is a major drawback for this approach. Unless there is a change in current thinking, bacterial and yeast cellulase production systems may still be the most feasible choices for future biofuel production.

## Abbreviations

BGl: β-glucosidase
CBH: Cellobiohydrolase
CBM: Carbohydrate-binding module
CBP: Consolidated bioprocessing
CMC: Carboxymethyl cellulose
EG: Endoglucanase
ER: Endoplasmic reticulum
RESS: Repression under secretion stress
Sec: Secretion protein
SNAREs: Soluble NSF (N-ethylmaleimide-sensitive factor) attachment receptor proteins
Tat: Twin-arginine transporter
TSP: Total soluble protein
